# Effects of early introduction of solid foods on nutrient intake in preterm infants during their 1st year of life: a secondary outcome analysis of a prospective, randomized intervention study

**DOI:** 10.3389/fnut.2023.1124544

**Published:** 2023-05-18

**Authors:** Melanie Gsoellpointner, Fabian Eibensteiner, Margarita Thanhaeuser, Robin Ristl, Bernd Jilma, Angelika Berger, Nadja Haiden

**Affiliations:** ^1^Department of Clinical Pharmacology, Medical University of Vienna, Vienna, Austria; ^2^Department of Pediatrics and Adolescent Medicine, Comprehensive Center for Pediatrics, Medical University of Vienna, Vienna, Austria; ^3^Center for Medical Statistics, Informatics and Intelligent Systems, Medical University of Vienna, Vienna, Austria

**Keywords:** complementary feeding, preterm infants, nutrient intake, iron intake, vitamin D intakes

## Abstract

Very low birth weight (VLBW) infants have higher nutritional needs even after hospital discharge. However, data concerning current nutrient intakes at different time points after the introduction of solid foods and whether dietary reference values are being met are scarce. To adress this issue, this secondary analysis of a prospective, two-arm interventional study in 177 VLBW infants 21 investigates dietary intake comparing early and late (early: 10–12 weeks corrected for gestational age, late: 16–18 weeks corrected for gestational age) introduction of standardized complementary food during the first year of life. Nutritional intake was assessed using self-reported monthly 3-day dietary records from 3 until 12 months, corrected for gestational age. The time point of the introduction of solid foods did not influence nutrient intake, but the early introduction of solids tended toward a higher proportional intake of protein and carbohydrates and a lower intake of fat as a percentage of total energy) during the 1st year of life, corrected for gestational age. The results of this study indicate that this standardized feeding concept was sufficient for zinc, calcium, and phosphorus intake. However, dietary iron and vitamin D intakes did not meet the recommendations. Thus, prolonged iron supplementation should be considered beyond the introduction of meat and vitamin D supplementation at least until 12 months, corrected for gestational age.

**Trial registration number:**

ClinicalTrials.gov: NCT01809548.

## 1. Introduction

The first 1,000 days of life, also known as “the window of opportunity,” represent a critical period of not only immense potential but also an immense vulnerability that can affect an infant's development and metabolic programming (1–3). The nutritional intake of very low birth weight (VLBW) infants requires specific attention, as preterm infants are assumed to have higher nutritional requirements even after being discharged from the hospital (4). After a certain period, breastmilk or formula alone is no longer sufficient to cover their nutritional needs. Thus, solid foods have to be gradually introduced into the infants' diet (5). Current evidence indicates that preterm infants are introduced to solid foods earlier than term infants (6, 7). However, dietary intakes at different time points after introducing solid foods and whether dietary intake reference values are met during the period of complementary feeding (CF) are unknown. Especially at the beginning of CF, there is a higher risk of nutritional imbalances due to changes in macronutrients and micronutrients (8). Notably, protein, iron, vitamin D, zinc, calcium, and phosphorus rank among the critical nutrients in preterm infants, as suboptimal intakes are associated with impaired growth (9), poor short- and long-term health outcomes, impaired neurodevelopment (10), and poor bone health (11). To ensure optimal growth in preterm infants, it is necessary to determine whether the time point of the introduction of solids is safe in terms of macronutrient intake. Protein intake plays a major role, as low protein intake is associated with undernutrition (12, 13), and high protein intake might increase the risk of obesity in later life (14, 15). Moreover, micronutrient supply during CF in preterm infants is an intensively debated topic due to the essential role of CF in physical growth and neuromotor development (16). However, it is unclear to what extent the timing of the introduction of solid foods affects micronutrient intake throughout the 1st year.

This study aimed to investigate the dietary macronutrient and micronutrient intake at two different time points after the introduction of solid foods in VLBW infants fed a standardized diet during the 1st year of life and whether the dietary intake meets current dietary reference values.

## 2. Methods

### 2.1. Study design

This is a secondary analysis of nutritional data collected during a prospective, randomized, two-arm intervention trial of VLBW infants with a birthweight below 1,500 g that were followed in the outpatient clinic of the Division of Neonatology, Department of Pediatrics, Medical University of Vienna from October 2013 until February 2020. Infants were randomized to an early CF group (introduction of complementary food between 10–12 weeks corrected for gestational age) or a late CF group (introduction of complementary food between 16–18 weeks corrected for gestational age) and fed a standardized feeding concept throughout the 1st year of life. Infants with a birth weight of <1,500 g were eligible to participate in the study. The exclusion criteria were any diseases that affect stable growth (i.e., Hirschsprung disease (17), chronic inflammatory bowel disease (18), bronchopulmonary dysplasia (19), necrotizing enterocolitis (NEC) with short bowel syndrome (20), any chromosomal aberrations, congenital heart disease (21), or major congenital birth defects). Information on the study design, sample size planning, and randomization process can be found in the primary outcome report of this trial (22).

The trial was approved by the ethics committee of the Medical University of Vienna (EK: 1744/2012) and registered on clinicaltrials.gov (NCT01809548). Written informed consent was obtained from at least one parent.

### 2.2. Study visits and diet

During the intervention period, study visits were scheduled in the neonatal outpatient clinic at the expected due dates of 6 weeks, 12 weeks, 6 months, and 12 months, corrected for gestational age. According to a standard operating procedure, anthropometric data (body weight, length, and head circumference) were measured at the respective dates; these primary outcome data were published previously (22). In addition to formula or mother's milk, infants were fed a standardized age-dependent step-up CF concept consisting of five different boxes with manifold, preprepared complementary foods according to the infants' ability to tolerate textures and pieces. Beforehand, a nutritionist calculated a diet rich in vitamin D, iron, calcium, phosphorus, omega-3 fatty acids, zinc, and folic acid and compiled the food boxes, offering a varied range of flavors. Feeding boxes ranged from finely pureed fruits and vegetables in the scoop familiarization phase to coarser-textured menus extended by grains, meat, fish, and milk products in the later phases of CF. The commercially available, ready-to-use baby jar food was provided for free by Nestle^?^ company (Vienna, Austria). Parents were able to pick up the food boxes at any time and had to adhere to the diet for more than 80% of the day during the infants' 1st year of life, corrected for gestational age. To verify adherence to the diet, parents had to complete self-reported 3-day dietary records in each of the study months (23, 24).

### 2.3. Data collection and evaluation

This secondary analysis aimed to evaluate the dietary intake of critical nutrients in VLBW infants during the 1st year of life. Dietary intake was estimated using 3-day dietary records for 3 consecutive days, including 1 weekend day. Parents of the infants enrolled in the trial were instructed and trained by a nutritionist to make a log of a detailed food report listing each enteral intake once a month from 3 to 12 months, corrected for gestational age (M3-M12). Diet records were analyzed using a nutritionist using nutrient software (nut.s nutritional. software, Vienna, Austria) based on the German Nutrient Database and the Austrian Nutrient Table (Version II.3.1). In infants who were breastfed, the exact milk intake was unknown. Hence, the estimated average values of consumed mother's milk, published by Dewey KG et al. (25), were used. Infant formula was not provided by the study team. However, detailed information on the formula used had to be documented in each protocol. To ensure accurate nutrient analysis, recipes for all infant formulas were requested by the distinct manufacturers, and changes in formulations were considered for calculation. Body weight at the respective date of the dietary record was used to calculate protein, vitamin D, and iron intake based on the amount per kg of body weight. For the remaining months in which no measurements were conducted, body weight was calculated based on the daily increase in body weight between the closest two measurements. Infants received 650 IU/d of vitamin D3 supplementation until 1 year, corrected for gestational age, and 2–3 mg/kg/d iron (Ferrum Hausmann, iron oxide polymaltose complex, Vifor^?^ France, Paris, France) until meat was fed regularly.

Furthermore, a multivitamin preparation (vitamins A, E, D3, B1, B2, B6, C, niacin, and pantothenic acid; Multibionta?Merck Selbstmedikation GmbH, Darmstadt, Germany) was given until 1 year, corrected for gestational age. The exact dosage and the respective start and end dates of all supplements were documented and used for daily intake calculation. Dietary intake is defined as oral intake solely from foods, whereas total intake combines dietary and supplemental intake.

### 2.4. Outcome parameters

The primary outcome of this analysis was protein intake (g/kg/d) throughout the 1st year of life, corrected for gestational age. Secondary outcomes included macronutrient distribution, i.e., protein, fat, and carbohydrates as a percentage of total energy, energy, vitamin D, iron, zinc, calcium, and phosphorus intake. If possible, nutrient intake was further compared with currently available intake recommendations for preterm infants [protein (8), vitamin D, and iron (26)]. If there were no specific reference values for preterm infants, those of term infants were used for comparison (energy, macronutrient distribution, zinc, calcium, and phosphorus) (27).

### 2.5. Statistical analysis

Nutrient intake was compared between the early and late introduction of solid foods. Data were analyzed according to the per-protocol principle. Patients with less than 80% adherence to the study food were excluded from the analysis. Furthermore, subjects who moved, were lost to follow-up, withdrew informed consent, or had no dietary record were also removed from data analysis. However, dietary protocols for subjects who moved or withdrew informed consent before being excluded from the analysis were included. Per-protocol statistical analysis of all primary and secondary outcomes was assessed using the linear mixed-effects models, accounting for randomization group, sex, gestational age, and nutrition at discharge as covariates, with a random intercept to account for possible correlations between siblings of multiple births. Marginal means (i.e., averaged across covariates) for the two groups were calculated from the linear mixed models according to standard errors and *p*-values for the null hypothesis of no between-group difference. Graphical analyses represent estimated marginal means and standard errors (error bars). *P*-values of <0.05 were considered statistically significant. As an additional analysis, the *p*-values for between-group comparisons of the same nutrient at different time points were adjusted using the Bonferroni method for results that were statistically significantly unadjusted, and the adjusted *p*-values (*p*-adj) are given in the text. Statistical analysis was conducted using RStudio Core Team (2022) (28).

## 3. Results

### 3.1. Participants and baseline characteristics

In total, 177 infants were included in the study, of whom 89 of them were randomized to the early feeding group and 88 to the late feeding group. The per-protocol cohort included 83 infants in the early group and 82 infants in the late group. Six infants (three in each group) were excluded from the analysis as they did not adhere to the study protocol. The parents of two infants withdrew consent (one in each group). Two infants in the late group moved prior to CF introduction; hence, no dietary protocols were available. Moreover, two infants in the early group were excluded from the analysis because of missing data on the variable “nutrition at discharge,” which was integrated into the statistical model.

Demographic parameters, as well as neonatal, obstetric, and parental parameters, were previously published (22, 29). The mean gestational age at birth was 27 + 1/7 in the early group and 27 + 2/7 in the late group, whereas the mean birthweight was 929 (SD ± 248) g and 932 (SD ± 256) g, respectively.

### 3.2. Dietary records

Dietary records valid for per-protocol analysis varied among groups and the respective time points, ranging from 66% (55/83) to 83% (69/83) in the early group and from 64% (53/82) to 77% (63/82) in the late group (Table 1).

**Table 1:** Dietary Protocols valid for per-protocol analysis.

**Months of life corrected for gestational age**	**Early group (*n* = 83)**	**Late group (*n* = 82)**	**Total (*n* = 165)**
3	69	56	125
4	58	60	118
5	66	57	123
6	64	62	126
7	56	57	113
8	58	63	121
9	60	55	115
10	58	54	112
11	55	54	109
12	58	53	111

### 3.3. Protein

**Figure 1:**
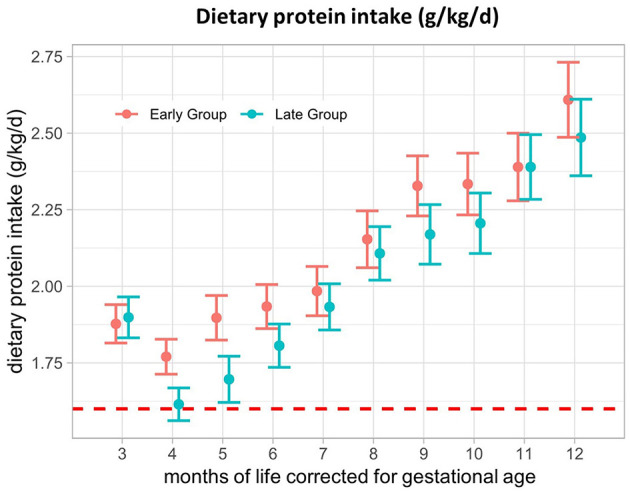
Dietary protein intake (g/kg/d) from 3–12 months of life, corrected for gestational age. The red dotted line represents the reference value for protein intake in preterm infants (1.6 g/kg/d). Presented as the estimated marginal mean and standard error of the linear mixed-effects models.

The results of the primary outcome of dietary protein intake (g/kg/d) are shown in Figure 1. We observed no significant difference in protein intake between the early and late groups throughout the 1st year of life, corrected for gestational age. In M3, protein intake was 1.88 (SE ± 0.06) g/kg/d in the early group and 1.90 (SE ± 0.07) g/kg/d in the late group and increased up to 2.61 (SE ± 0.12) g/kg/d and 2.49 (SE ± 0.13) g/kg/d in M12, respectively. Immediately after CF introduction, protein intake dropped in both groups, with a greater decline in the late group and a rebound in M5. Participants generally had a mean protein intake above the recommendations (6–12 months uncorrected age: 1.6 g/kg/d) (8) at any of the investigated time points, with an excessive intake of up to 2.61 g/kg/d in the second half of the intervention period (Table 2).

**Table 2:** Nutrient intake—Early vs. late introduction of complementary feeding.

**Months corrected age**		**3**		**Intake Recommendation**		**4**		**5**	
**Group**		**Early (*****n*** = **69)**	**Late (*****n*** = **56)**	**0-3 months**		**Early (*****n*** = **58)**	**Late (*****n*** = **60)**	**Early (*****n*** = **66)**	**Late (*****n*** = **57)**
Protein	g/kg/d	1.88 (0.06)	1.90 (0.07)	1.6 g/kg/d		1.77 (0.06)	1.61 (0.05)	1.90 (0.07)	1.70 (0.08)
Energy	kcal/d	500 (11)	497 (11)	500–550 kcal/d		534 (14)	508 (13)	576 (17)	557 (18)
Protein	% of energy	7.7 (0.2)	8.2 (0.2)	Not available		7.6 (0.1)	7.6 (0.1)	**8.2 (0.2)** ^*^	**7.7 (0.2)** ^*^
Fat	% of energy	**46.6 (0.4)** ^*^	**48.3 (0.4)** ^*^	45–50% of energy		44.6 (0.5)	45.9 (0.5)	**40.4 (0.7)** ^*^	**43.1 (0.8)** ^*^
Carbohydrates	% of energy	**45.7 (0.4)** ^*^	**43.6 (0.4)** ^*^	Not available		47.9 (0.5)	46.5 (0.5)	**51.4 (0.7)** ^*^	**49.2 (0.7)** ^*^
Iron	mg/kg/d	0.77 (0.05)	0.73 (0.05)	2–3 mg/kg/d		0.73 (0.04)	0.69 (0.04)	0.80 (0.04)	0.72 (0.04)
Iron total	mg/kg/d	4.0 (0.2)	3.6 (0.2)			3.9 (0.2)	3.4 (0.2)	3.5 (0.2)	3.2 (0.2)
Vit D diet	IU/d	279 (23)	297 (25)	800–1000 IU/d		272 (21)	296 (19)	306 (21)	303 (22)
Vit D total	IU/d	948 (28)	960 (30)			923 (28)	955 (26)	967 (28)	978 (29)
Calcium	mg/d	342 (15)	371 (16)	220 mg/d		350 (15)	348 (14)	388 (19)	376 (19)
Phosphorus	mg/d	201 (9)	210 (10)	120 mg/d		220 (10)	212 (9)	251 (13)	241 (13)
**Zinc**	mg/d	3.7 (0.2)	3.9 (0.2)	1.5 mg/d		3.9 (0.2)	4.0 (0.2)	4.3 (0.2)	4.1 (0.2)
**Months corrected age**		**6**		**7**		**8**		**9**	
**Group**		**Early (*****n*** = **64)**	**Late (*****n*** = **62)**	**Early (*****n*** = **56)**	**Late (*****n*** = **57)**	**Early (*****n*** = **58)**	**Late (*****n*** = **63)**	**Early (*****n*** = **60)**	**Late (*****n*** = **55)**
Protein	g/kg/d	1.93 (0.07)	1.81 (0.07)	1.98 (0.08)	1.93 (0.08)	2.15 (0.09)	2.11 (0.09)	2.33 (0.10)	2.17 (0.10)
Energy	kcal/d	602 (16)	605 (16)	633 (19)	638 (18)	672 (21)	689 (20)	737 (24)	710 (24)
Protein	% of energy	8.7 (0.2)	8.3 (0.2)	9.2 (0.2)	8.7 (0.2)	**9.7 (0.2)** ^*^	**9.1 (0.2)** ^*^	10.0 (0.2)	9.5 (0.2)
Fat	% of energy	37.7 (0.8)	38.1 (0.8)	33.7 (0.9)	35.5 (0.9)	32.4 (0.9)	34.0 (0.9)	32.2 (1.1)	32.4 (1.1)
Carbohydrates	% of energy	53.6 (0.7)	53.6 (0.7)	57.1 (0.9)	55.8 (0.8)	57.9 (0.8)	56.9 (0.8)	57.7 (1.0)	58.0 (1.1)
Iron	mg/kg/d	0.83 (0.04)	0.79 (0.04)	0.81 (0.05)	0.88 (0.05)	0.89 (0.05)	0.91 (0.05)	0.95 (0.05)	0.90 (0.05)
Iron total	mg/kg/d	3.2 (0.2)	2.8 (0.2)	2.2 (0.2)	2.1 (0.2)	1.8 (0.2)	1.9 (0.2)	1.8 (0.2)	1.8 (0.2)
Vit D diet	IU/d	299 (19)	308 (18)	276 (21)	304 (19)	304 (20)	308 (19)	322 (21)	305 (21)
Vit D total	IU/d	963 (26)	975 (25)	946 (34)	952 (32)	972 (32)	958 (30)	960 (37)	930 (37)
Calcium	mg/d	408 (17)	405 (16)	424 (19)	424 (18)	473 (23)	451 (21)	520 (24)	483 (24)
Phosphorus	mg/d	269 (11)	268 (11)	302 (12)	279 (11)	329 (14)	322 (13)	371 (13)	355 (13)
Zinc	mg/d	4.6 (0.2)	4.5 (0.2)	4.4 (0.2)	4.8 (0.2)	4.9 (0.2)	5.1 (0.2)	5.5 (0.2)	5.2 (0.2)
**Months corrected age**		**10**		**11**		**12**		**Intake Recommendations**	
**Group**		**Early (*****n*** = **58)**	**Late (*****n*** = **54)**	**Early (*****n*** = **55)**	**Late (*****n*** = **54)**	**Early (*****n*** = **58)**	**Late (*****n*** = **53)**	**4–12 months**	
Protein	g/kg/d	2.33 (0.10)	2.21 (0.10)	2.39 (0.11)	2.39 (0.11)	2.61 (0.12)	2.49 (0.13)	1.6 g/kg/d	
Energy	kcal/d	755 (24)	732 (24)	759 (26)	776 (25)	814 (27)	827 (27)	600–700 kcal/d	
Protein	% of energy	10.2 (0.2)	9.8 (0.2)	10.7 (0.2)	10.4 (0.2)	11.4 (0.3)	10.7 (0.3)	Not available	
Fat	% of energy	32.1 (0.9)	31.5 (0.9)	30.3 (0.9)	30.8 (0.9)	30.4 (0.8)	29.5 (0.9)	35–45 % of energy	
Carbohydrates	% of energy	57.8 (0.8)	58.7 (0.8)	59.0 (0.8)	58.8 (0.8)	58.2 (0.8)	59.8 (0.8)	Not available	
Iron	mg/kg/d	0.95 (0.05)	0.95 (0.05)	0.96 (0.05)	0.92 (0.05)	0.98 (0.05)	0.92 (0.06)	2–3 mg/kg/d	
Iron total	mg/kg/d	1.4 (0.2)	1.7 (0.2)	1.5 (0.2)	1.5 (0.2)	1.5 (0.2)	1.6 (0.2)		
Vit D diet	IU/d	305 (19)	310 (19)	302 (20)	276 (19)	318 (21)	275 (21)	800–1,000 IU/d	
Vit D total	IU/d	865 (49)	857 (48)	813 (54)	785 (51)	694 (56)	660 (58)		
Calcium	mg/d	577 (26^*^)	501 (26^*^)	601 (30)	569 (28)	656 (31)	613 (31)	330 mg/d	
Phosphorus	mg/d	419 (16^*^)	361 (16^*^)	446 (21)	430 (20)	511 (23)	472 (23)	300 mg/d	
Zinc	mg/d	5.7 (0.2)	5.4 (0.2)	5.7 (0.2)	5.7 (0.2)	5.9 (0.2)	6.0 (0.2)	2.5 mg/d	

The data are presented as the estimated marginal mean and standard error of the linear mixed-effects models of the per-protocol study population. Significant differences before correction for multiple testing (Bonferroni) were marked with^*^.

### 3.4. Energy intake and macronutrient distribution

**Figure 2:**
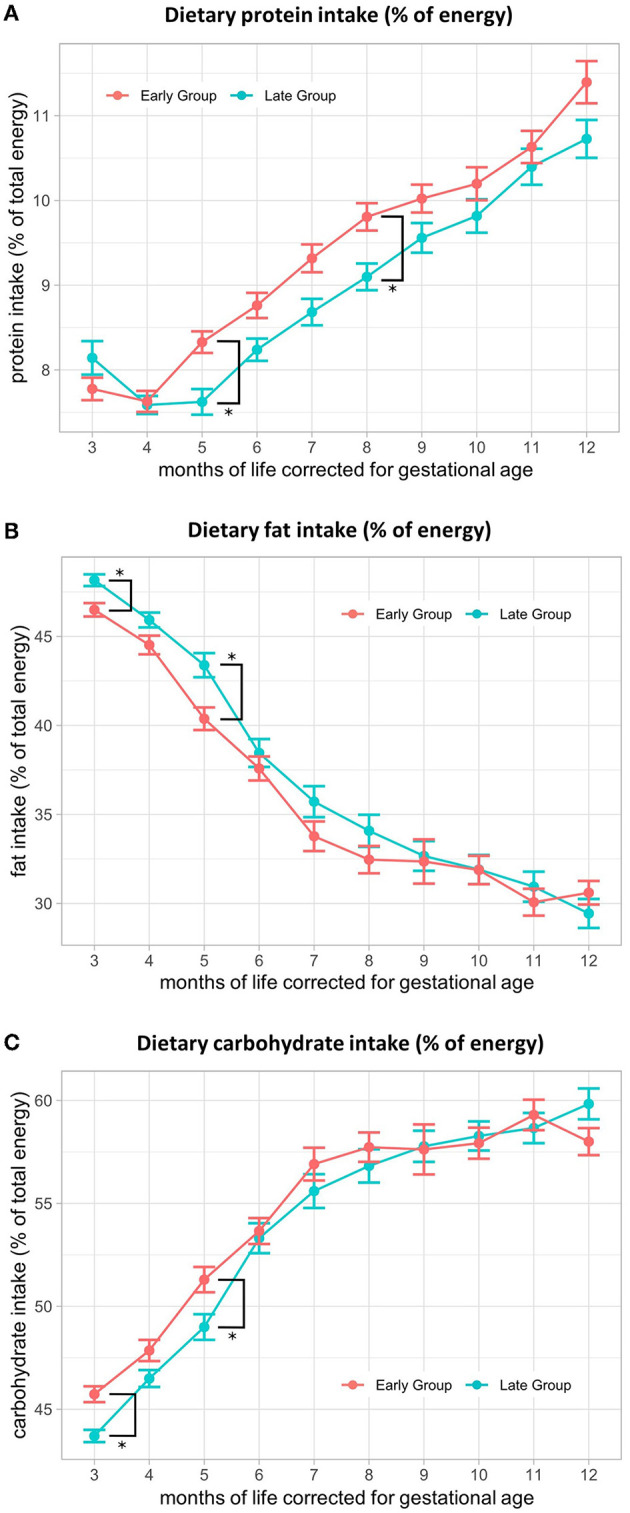
Macronutrient distribution (% of energy) from 3–12 months of life, corrected for gestational age. Comparison of **(A)** protein intake in % of energy, **(B)** fat intake in % of energy, and **(C)** carbohydrate intake in % of energy between early and late CF introduction from 3 to 12 months, corrected for gestational age. Presented as the estimated marginal mean and standard error of the linear mixed-effects models.

There was no significant difference in energy intake (kcal/d) between the early and late groups at any of the investigated time points (Table 2). At M3, mean energy intake was 500 (SE ± 11) kcal/d in the early group and 497 (SE ± 11) kcal/d in the late group, increasing to 814 (SE ± 27) kcal/d and 827 (SE ± 27) kcal/d in M12, respectively. Macronutrient distribution varied in the 1st months after the introduction of solid foods between the groups. The percentage of protein intake from total energy was significantly higher in M5 [early: 8.2% (SE ± 0.2), late: 7.7% (SE ± 0.2); *p* = 0.03; *p*-adj = 0.27] and M8 (early: 9.7% (SE ± 0.2), late: 9.1% (SE ± 0.2); *p* = 0.03; *p*-adj = 0.27) in the early feeding group, with a persisting trend toward higher intake when compared to the late group (Figure 2A). Fat intake in the percentage of total energy was significantly higher in the late group in M3 (early: 46.6% (SE ± 0.4), late: 48.3% (SE ± 0.4); *p* = 0.002; *p*-adj = 0.02) and M5 (early: 40.4% (SE ± 0.7), late: 43.1% (SE ± 0.8), *p* = 0.01; *p*-adj = 0.12) (Figure 2B), whereas the percentage of carbohydrate intake from total energy was higher in the early group in M3 (early: 45.7% (SE ± 0.4), late: 43.6% (SE ± 0.4); *p* = 0.0002; *p*-adj = 0.002) and M5 (early: 51.4% (SE ± 0.7), late: 49.2% (SE ± 0.7); *p* = 0.03; *p*-adj = 0.28) (Figure 2C). Upon correction for multiple testing, proportional fat intake and carbohydrate intake at M3 remained significant. A comparison of the fat intake data from this study with term infant recommendations for energy distribution (0–3 months: 45–50%, 4–12 months: 35–45%) (27) showed that fat intake (% of energy) was below the recommendations from M7 in the early group and M8 in the late group onwards (Table 2).

### 3.5. Iron

**Figure 3:**
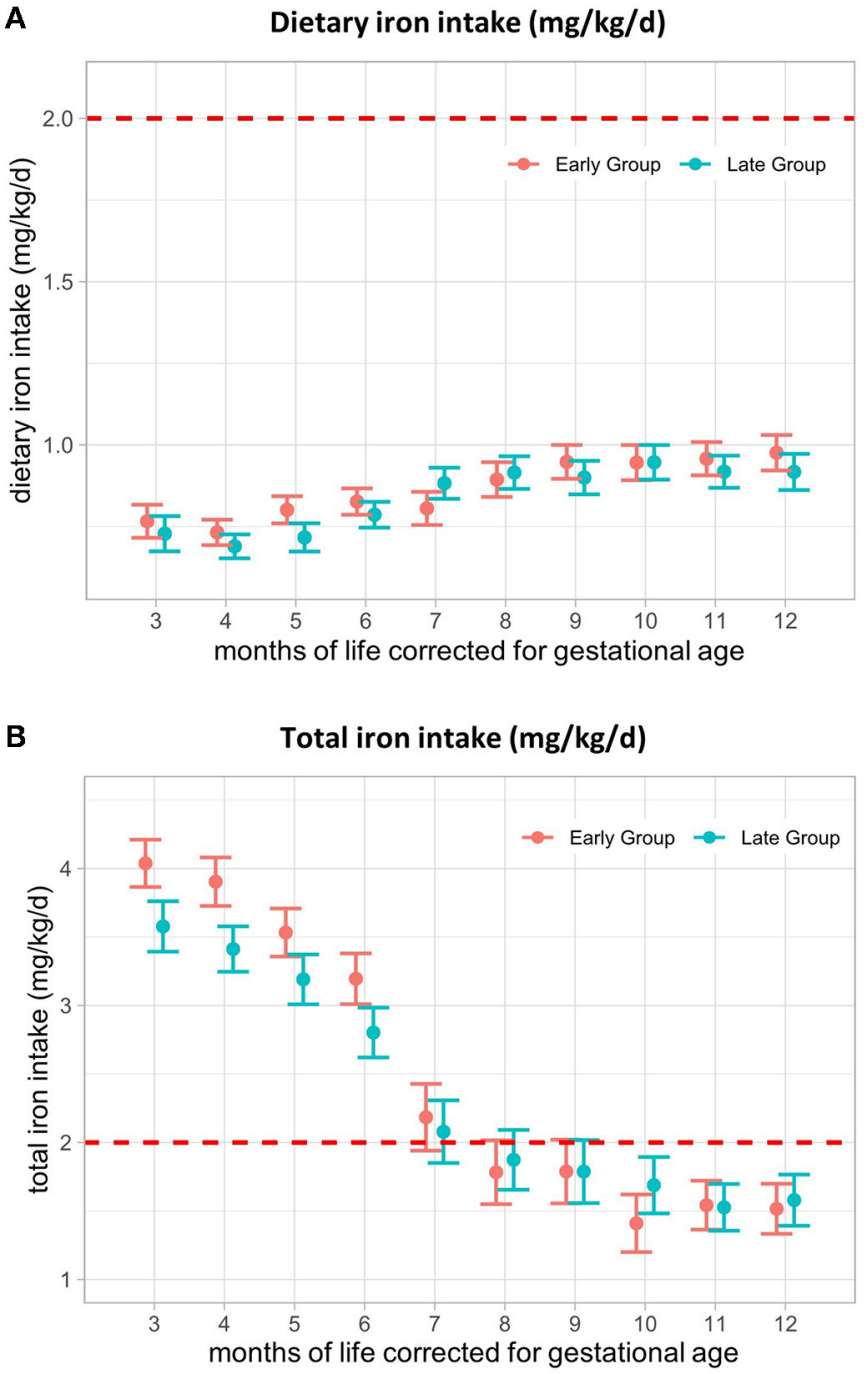
Iron intake (mg/kg/d) from 3–12 months of life, corrected for gestational age. **(A)** Dietary iron intake and **(B)** total iron intake from 3–12 months, corrected for gestational age in mg/kg/d. The red line represents the lower limit of ESPGHAN iron intake recommendations for VLBW infants (2–3 mg/kg/d until 6–12 months, depending on the diet). Presented as the estimated marginal mean and standard error of the linear mixed-effects models. Total = supplemental + dietary intake.

We found no difference in mean dietary iron intake between the early and late groups during the intervention period (Figure 3A). In M3, mean dietary iron intake was 0.77 (SE ± 0.05) mg/kg/d in the early group and 0.73 (SE ± 0.05) mg/kg/d in the late group. After an initial decrease, iron intake increased again, with levels reaching 0.98 (SE ± 0.05) mg/kg/d and 0.92 (SE ± 0.06) mg/kg/d in M12, respectively (Figure 3A), At M3, total iron intake was 4.0 (SE ± 0.2) mg/kg/d in the early group and 3.6 (SE ± 0.2) mg/kg/d in the late group and decreased to 1.5 (SE ± 0.2) mg/kg/d and 1.6 (SE ± 0.2) mg/kg/d at M12, respectively (Figure 3B). From 8 months, corrected for gestational age onwards, total iron intake dropped below the intake recommended by ESPGHAN (2–3 mg/kg/d until 6–12 months of age, depending on the diet) (26). This was mainly caused by the termination of additional iron supplementation for most of the infants. Dietary iron intake by CF could not compensate for iron intake administered by iron supplementation, resulting in a very low intake in the second half of the intervention period (Table 2).

### 3.6. Vitamin D

**Figure 4:**
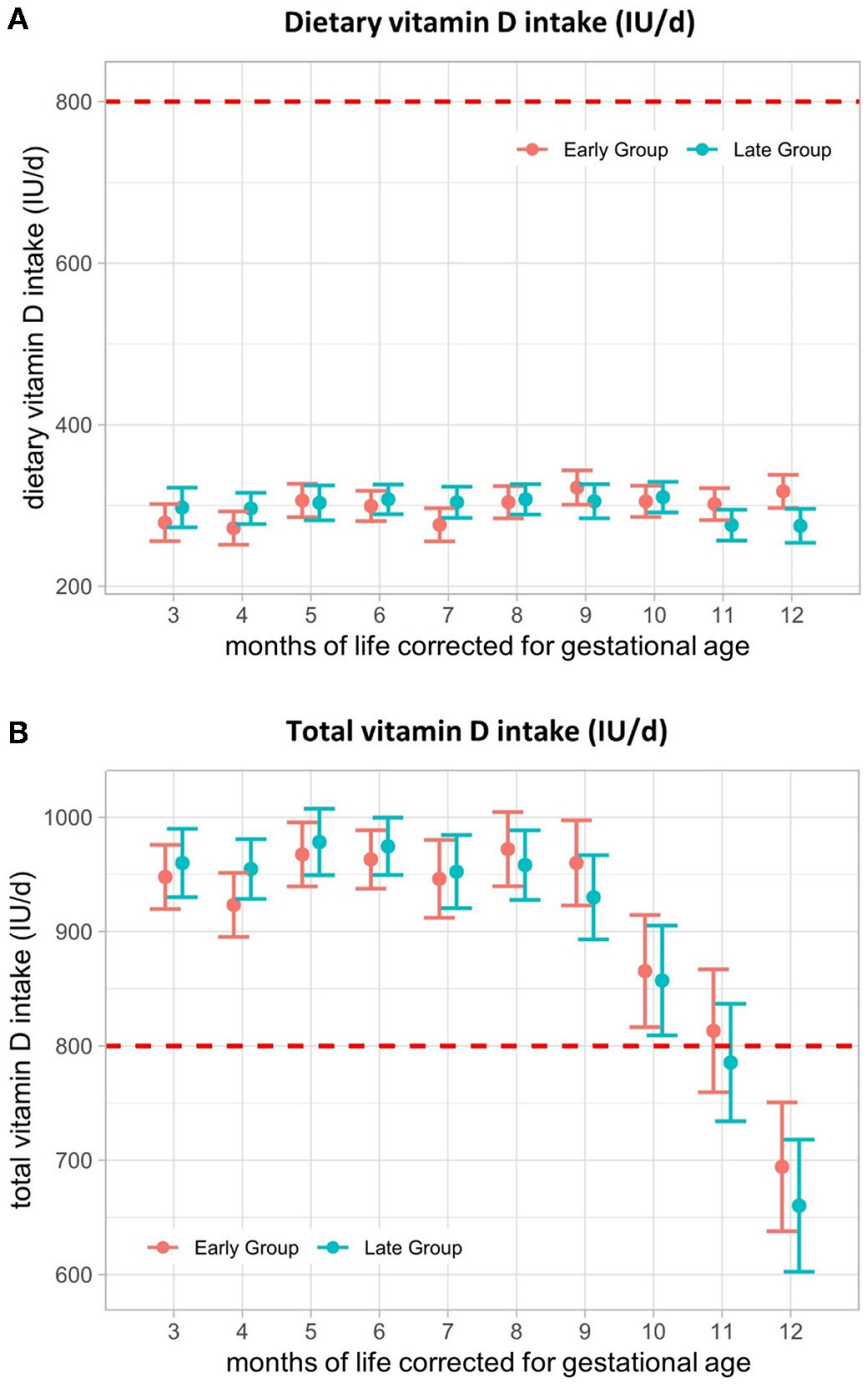
Vitamin D intake (IU/d) from 3–12 months of life, corrected for gestational age. **(A)** Dietary vitamin D intake and **(B)** total vitamin D intake from 3–12 months, corrected for gestational age in IU/d. The red line represents the lower limit of ESPGHAN vitamin D recommendations for VLBW infants during the 1st year of life. Presented as the estimated marginal mean and standard error of the linear mixed-effects models. Total = supplemental + dietary intake.

The mean dietary vitamin D intake did not differ between the early and late groups, ranging from a minimum of 272 (SE ± 21) IU/d to a maximum of 322 (SE ± 21) IU/d (Figure 4A) and was not influenced by CF introduction. Dietary intake together with supplemental vitamin D intake was not different between the groups and remained within the range of the recommendations (800–1,000 IU/d during the 1st year of life (26) until 10 months, corrected for gestational age (M10- early: 865 (SE ± 49) IU/d, late: 857 (SE ± 48) IU/d) (Figure 4B). From M11, total vitamin D intake fell below recommendations in the late group and from M12 in the early group, mainly related to the termination of vitamin D supplements (Table 2).

### 3.7. Calcium

Dietary calcium intake did not differ between the groups throughout the 1st year, corrected for gestational age, except for M10 (early: 577 (SE ± 26) mg/d, late: 501 (SE ± 26) mg/d; *p* = 0.04; *p*-adj = 0.45). After multiple testing adjustments, no significance was detected anymore. Mean dietary calcium intakes ranged from 342 (SE ± 15) mg/d in M3 to 656 (SE ± 31) mg/d in M12 in the early group and from 371 (SE ± 16) mg/d in M3 up to 613 (SE ± 31) mg/d in M12 in the late group. Plotting our data against the current recommendations for calcium intake for term infants (220 mg/d for infants from 0–3 months and 330 mg/d for 4–12 months) (27), we found that both groups met these recommendations at all of the investigated time points (Table 2).

### 3.8. Phosphorus

Dietary phosphorus intake only differed significantly in M10 between the early and the late groups [early: 419 (SE ± 16) mg/d, late: 361 (SE ± 16) mg/d; *p* = 0.01; p-adj = 0.14]. After multiple testing adjustments, no significance was detectable. Mean dietary phosphorus intake increased from 201 (SE ± 9) mg/d in the early group and 210 (SE ± 10) mg/d in the late group at the beginning to 511 (SE ± 23) mg/d and 472 (SE ± 23) mg/d at the end of the intervention period. Dietary reference values (0–3 months: 120 mg/d; 4–12 months: 300 mg/d) (27) were met only at the very beginning of weaning (M3) and from M8 onwards. Dietary phosphorus intake was below 300 mg/d in both groups in M4-M6 and M7 in the late group (Table 2).

### 3.9. Zinc

The time point of the introduction of solid foods did not influence dietary zinc intakes, which were above the reference values (0–3 months: 1.5 mg/d; 4–12 months: 2.5 mg/d) of term infants at all investigated time points in both groups (Table 2). Mean dietary intakes ranged from 3.7 (SE ± 0.2) mg/d in the early group and 4.0 (SE ± 0.2) mg/d in the late group to 5.9 (SE ± 0.2) mg/d and 6.0 (SE ± 0.2) mg/d, respectively (Table 2).

## 4. Discussion

This is a secondary outcome analysis of a randomized intervention trial investigating the nutritional intake after the introduction of standardized CF in VLBW infants at two different time points. This study showed that the time point of the introduction of solid foods had no impact on protein intake or any other investigated nutrients. The early introduction of CF was associated with a higher proportion of protein and carbohydrate intake and a lower fat intake at the beginning of CF. Dietary intake recommendations were met for zinc, calcium, and phosphorus but not for iron and vitamin D.

### 4.1. Protein intake and macronutrient distribution

The time point of the introduction of solid foods had no impact on dietary protein intake (g/kg/d) during the 1st year of life, corrected for gestational age. To date, only little is known about protein intake during CF in preterm infants and its relation to the time point of CF introduction. In the study by Mariott et al., published in 2003, preterm infants were randomized to a “preterm weaning strategy” (PWS) group or a control group. Infants in the PWS group started eating semisolid foods at 13 weeks of postnatal age, whereas infants in the control group were introduced to solid foods after 17 weeks of postnatal age, provided that they weighed 3.5 and 5 kg, respectively. The authors reported a significantly higher mean growth rate of length/week in the PWS group and a significantly higher intake of total energy (PWS: 822 kcal/d, control: 728 kcal/d) and protein at 6 months, corrected for gestational age (PWS: 26.7 g/d, control: 23 g/d) but no differences at 12 months, corrected for gestational age. (30) In the study by Mariott et al., protein and energy intake were significantly higher, with a protein intake that was twice as high as ours. It is unclear how such a high intake could be achieved. However, the results are no longer universally applicable because the study was performed when post-discharge formula and breastmilk fortification were not available. Hence, solids were used for enhanced nutritional intake as other options were lacking. Optimal protein intake during CF in preterm infants is important, as insufficient protein intake can contribute to undernutrition (12, 13). The beginning of CF is especially vulnerable to the risk of nutritional imbalances due to the changes in macronutrients and micronutrients (8). Although protein intake immediately decreased after the introduction of CF, mean protein intake (g/kg/d) did not drop below the recommendations (1.6 g/kg/d) in both groups at any of the investigated time points, assuming that early and late introduction is adequate for the prevention of undernutrition in former preterm infants. However, protein intake exceeded the recommendations, with levels up to 2.61 (SE ± 0.1) g/kg/d in the second half of the 1st year, corrected for gestational age. There is a growing body of evidence supporting the hypothesis that excessive protein intake during the period of CF is associated with overweight and obesity during childhood in healthy-term infants (14, 15). Günther et al. (31) showed that a consistently high protein intake at the age of 12 months was related to a higher mean BMI standard deviation score and percentage of body fat at 7 years of age To prevent overweight and obesity later in life, it is suggested that protein intake not exceed 15% of energy (32). In our study, protein intake (% of energy) increased from 7.7% (SE ± 0.2) in the early group and 8.2% (SE ± 0.2) in the late group at M3 to 11.4% (SE ± 0.3) and 10.7% (SE ± 0.3) at M12, respectively. Thus, protein intake (% of energy) was within the safety range in both groups, avoiding the potential risk of wrong metabolic programming and adiposity in later life. Moreover, a higher protein intake may be advantageous for former preterm infants, especially those who have not reached catch-up growth by the time of the CF introduction. (33) The primary outcome of this study assessed length at 12 months, corrected for gestational age, which did not differ between the early and late groups. At 6 months, corrected for gestational age, the early group had significantly higher weight z-scores (22). A recent study demonstrated an association between high proportional protein intake and rapid weight gain in a dose-dependent manner during the CF period (34). Thus, a higher proportional protein intake (% of total energy) in the early group at the beginning of the weaning period might have contributed to higher weight z-scores at 6 months, corrected for gestational age. The results must be interpreted cautiously, as no significance was detected after correction for multiple testing. Furthermore, the study was not powered to detect a significant difference in nutrient intake between groups, and dietary records were not available for all patients enrolled in this study.

Furthermore, a proportional fat intake of 35–45% during the period of CF is suggested (27). The late group had a significantly higher fat intake compared to the early group at the beginning of CF. To date, no data on the proportional fat intake during CF and later health outcomes in former preterm infants is available. Concern has been raised that a higher fat intake is associated with obesity in adults (32). However, there is increasing evidence that, in term infants, a higher fat intake during CF is not associated with overweight later in life. Indeed, the results of the study by Rolland–Cacher et al. demonstrated that a low fat intake at 2 years might lead to an increased risk of obesity and leptin resistance in adulthood (35). With respect to this, a late CF introduction might be more favorable to preventing obesity during childhood. However, mean proportional fat intake was below the reference values in both groups in the second half of the intervention period. Thus, there is a general need to improve proportional fat intake to optimize growth and minimize adverse health outcomes later in life. It must be considered that not only fat quantity but also quality is of critical importance. A high intake of trans fatty acids is associated with increased inflammation and adverse effects on somatic development and should be avoided during CF (36). Polyunsaturated fatty acids, i.e., n-3 and n-6 fatty acids, are important for growth, neurodevelopment, and immune function (37). Thus, solid foods that are rich in polyunsaturated fatty acids, such as fish and vegetable oils, e.g., soybean and rapeseed oil, should be offered more frequently (38). However, further studies on proportional quantities of macronutrients during CF with respect to growth parameters and later health outcomes are needed.

### 4.2. Iron intake

In this study, the mean dietary iron intake did not differ between the early and late groups. In contrast, Kattelman et al. (39) reported that mean iron intake was greater when complementary foods were introduced early. The study authors concluded that the higher iron intake was likely due to the greater consumption of iron-fortified cereals as the first complementary food. In our study, infants mainly received pureed fruits and vegetables as their first solids, which provide only small amounts of iron and might explain the deficiency in dietary iron intake in both groups. Finn et al. showed that infants that consumed iron-fortified cereals from 6 to 18 months had significantly higher levels of iron compared to non-users, suggesting that this could be a strategy to reduce iron deficiency (40). Generally, dietary iron intake was low, with levels remaining below 1 mg/kg/d throughout the intervention period. Thus, CF failed to adequately improve dietary iron intake. This is in line with current literature that shows that up to 60% of infants have iron intake levels below the estimated average requirements from 6 to 36 months in some European countries, including Austria. (41). VLBW infants are even more prone to iron deficiency than term infants (42). Iron requirements cannot be met solely by dietary sources, emphasizing the need for iron supplementation in this vulnerable cohort. Although standard iron supplementation was given until meat was fed regularly, as suggested by ESPGHAN (43, 44), the mean total iron intake fell below the recommendations in the second half of the 1st year, corrected for gestational age. Data on iron status from this study had already been published previously (29). At 6 months, corrected for gestational age, 6% of infants in the early group and 8% in the late group developed iron deficiency (defined as ferritin <12 μg/L). The incidence of iron deficiency was significantly higher in the early feeding group at 12 months, corrected for gestational age (early: 13%, late: 2%). Because dietary iron intake was not statistically different and total iron intake was even higher in the early group at the beginning of CF, we assumed that the difference in iron deficiency between the groups may result from other factors, such as heme and none-heme iron composition, host-related factors, and potential individually different iron requirements, rather than overall iron intake. (45) Regardless of the timepoint of CF introduction, there is a need to improve iron intake and status in VLBW infants during the 1st year of life. This could be achieved by prolonged iron supplementation, nutrition counseling, and parental education on dietary sources of iron, as well as by improved compliance with iron supplementation and consequent monitoring of iron status.

### 4.3. Vitamin D intake

The results of this study showed that the timepoint of the introduction of solids had no influence on dietary vitamin D intake and that vitamin D intake did not change and improved throughout the 1st year of life, corrected for a term with constantly low intake levels ranging from 269 (SE ± 21) IU/d to 325 (SE ± 21) IU/d (see Table 2). These findings are consistent with previous literature that indicates that vitamin D requirements cannot be met solely by dietary sources during the 1st year of life (46). Infants received 650 IU/d of vitamin D3 supplementation, and the total vitamin D intake was within the recommended range of 800–1000 IU/d (ESPGHAN) until 10 months, corrected for gestational age. Although the recommendations for vitamin D intake were met, 67% in the early group and 49% in the late group developed vitamin D deficiency (serum 25-OH-vitamin D levels < 50 nmol/L) at 6 months, corrected for gestational age (47). The incidence of vitamin D deficiency was even higher at 12 months, corrected for gestational age (early: 89%, late: 81%) (47), suggesting that vitamin D requirements in preterm infants might be higher than previously assumed. Hence, vitamin D supplementation dose should be reconsidered, and factors affecting vitamin D bioavailability and absorption efficiency [i.e., food matrix, its interaction with other fat-soluble compounds, age, genetic variation, and disease (48)] should be further addressed to optimize vitamin D intake during CF in former VLBW infants. In the present study, the parents often stopped supplementation before 12 months, corrected for gestational age, further explaining low vitamin D serum levels at the end of the 1st year as dietary intake was insufficient. We suggest continuing vitamin D supplementation at least until 12 months after correction for gestational age and monitoring vitamin D status to adapt therapy if necessary.

### 4.4. Calcium and phosphorus intake

Dietary calcium and phosphorus intake were not influenced by the time point of the introduction of solid foods. Calcium intake was found to be within the reference values of term infants throughout the whole study period. However, whether these reference values are appropriate for use in former preterm infants is questionable because calcium absorption is compromised due to poor gastrointestinal tolerance and motility in preterm infants (49). Serum calcium levels at 6 weeks, 6 months, and 12 months, corrected for gestational age, were within the normal range, and no case of deficiency was reported (47), assuming that calcium intake was sufficient. Dietary phosphorus intake was below the recommendations, mainly in the transition phase from exclusive breastfeeding or formula feeding to solid foods from 5 to 7 months, corrected for gestational age. Phosphorus intake mainly derives from meat, fish, seafood, dairy products, seeds, whole grains, and nuts, all foods that are rather introduced later in the period of CF, thus explaining the shortfall during the respective months (50). Low dietary intakes of phosphorus may affect the optimal growth of infants and the deposition of lean body mass, as phosphorus is an important component of body tissue (51). Furthermore, inadequate intakes of phosphorus and calcium contribute to the etiology of metabolic bone disease in premature infants. Biomarkers suggestive of the diagnosis of metabolic bone disease are hypophosphatemia and high levels of alkaline phosphatase (52). Phosphorus and alkaline phosphate serum levels were within the reference range (47), assuming that intake levels were adequate to prevent metabolic disease.

### 4.5. Zinc intake

Zinc is a critical nutrient, especially in the early stages of life, as it serves many cellular processes (53). In our study, zinc intake was not influenced by the timepoint of introduction, and intake recommendations for term infants were met throughout the intervention period. Still, it is unclear whether the needs of premature infants with corrected ages correspond to those of mature infants. Preterm infants have higher zinc requirements in the early postnatal phases (54). However, no data regarding zinc requirements during the weaning period in former preterm infants exist. Because we did not measure serum zinc concentration, it was impossible to assess whether dietary zinc intakes efficiently prevent deficiency. Thus, the actual zinc requirements of former VLBW infants during the 1st year of life must be addressed further.

### 4.6. Study strengths and limitations

The standardized CF concept represents the major strength of this study, as it enabled exact calculations of nutritional intake in VLBW infants for the first time. The food provided in this study was commercially available baby jar food. Thus, the results of this study are generally applicable to preterm infants that are fed preprepared complementary food in Austria. Another strength of this study is the precise calculation of intakes from infant formula, as changes in formulations were considered for calculation. However, as this is a secondary outcome analysis of a randomized controlled trial, it was not powered to detect a difference in nutrient intake between study groups. A baseline imbalance in birthweight and gestational age occurred during study recruitment after an interim analysis in July 2017. Infants in the early group had a significantly lower birthweight and a significantly lower gestational age compared to the late group (22). Therefore, the randomization process was switched to a baseline adaptive randomization design with additional stratification according to birth weight. However, the products of the standardized solid food, their nutritional content, and the infant's nutritional intake remained the same over the whole study period. Hence, it is very unlikely that the baseline adaptive randomized design influenced any outcome parameter of this analysis. Another limitation of this study was that mothers' milk intakes were calculated as mean intakes based on previous studies because exact intake data were not available. Previous reports reported that the levels of some nutrients, including vitamin A, B6, vitamin B12, fatty acids, zinc, vitamin D, and iron, are associated with maternal factors such as maternal diet or supplementation (55–57). Because this study did not investigate maternal dietary intakes, variabilities in human milk were not considered. Furthermore, it was previously described that various factors, such as medication, maternal endocrine disorders, and temporarily blocked ducts, may pose a potential risk for low milk production, affecting milk volume intakes (58). Since these factors were not considered, the data must be interpreted cautiously. To inform readers about the lack of confounding that might be due to differences in breast lactation, maternal baseline characteristics from the per-protocol population are provided in the Supplemental material section.

## 5. Conclusions

The time point of the introduction of solid foods did not have an impact on nutrient intake. However, early introduction leads to a higher proportional intake of protein and carbohydrates and a lower fat intake (percentage of total energy) at the beginning of weaning. Further studies on macronutrient distribution during CF with respect to growth parameters and later health outcomes are needed to ensure optimal growth without the risk of obesity or wrong metabolic programming later in the lives of former preterm infants. The results of this study indicate that this standardized feeding regime provided sufficient zinc, calcium, and phosphorus intake. However, dietary iron intake was low even after introducing iron-rich foods, and recommendations were not met in the second half of the 1st year, corrected for gestational age. Therefore, prolonged iron supplementation should be considered, as iron intake solely from dietary sources is insufficient. Furthermore, dietary intake of vitamin D was insufficient to meet the recommendations throughout the 1styear of life, highlighting the importance of vitamin D supplementation until at least 12 months, corrected for gestational age. This study adds to our understanding of the dietary intake of critical nutrients during the complementary feeding period in VLBW infants, which is crucial in preventing both over- and under-supply and thus optimizing post-discharge nutritional management in this vulnerable cohort.

## Data availability statement

The datasets presented in this article are not readily available because data requestors will need to sign a data access agreement and in keeping with patient consent for secondary use, obtain ethical approval for any new analyses. The study protocol and the individual participant data that underlie the results reported in this article, after deidentification, are available upon request from the corresponding author 6 months after publication. Researchers will need to state the aims of any analyses and provide a methodologically sound proposal. Requests to access the datasets should be directed to nadja.haiden@meduniwien.ac.at.

## Ethics statement

The studies involving human participants were reviewed and approved by Ethics Committee of the Medical University of Vienna (EK:1744/2012, date of approval 10 January 2013) and registered on clinicaltrials.gov (https://clinicaltrials.gov, NCT01809548). Written informed consent to participate in this study was provided by the participants' legal guardian/next of kin.

## Author contributions

NH conceptualized the study. MG researched and analyzed the literature and wrote the manuscript, including interpretations. MG and MT contributed to the data acquisition for the study. MG and FE contributed to the formal analysis and visualization. NH, MT, FE, AB, RR, and BJ revised and edited the manuscript critically for intellectual content. All authors agree to be accountable for all aspects of the study, ensuring that questions related to the accuracy or integrity of any part of the study are appropriately investigated and resolved.
