# Supplementary material for: Effects of early introduction of solid foods on nutrient intake in preterm infants during their 1st year of life: a secondary outcome analysis of a prospective, randomized intervention study
Source: Front Nutr. 2023 May 18;10:1124544. doi: 10.3389/fnut.2023.1124544 (PMC10232899; doi:10.3389/fnut.2023.1124544)
Supplement: Supplementary file 1 [file Table_1.DOCX]

**SUPPLEMENTAL MATERIAL**

1. **Maternal baseline characteristics**

Maternal baseline characteristics are reported from the per-protocol population in the early and late complementary feeding group. Categorical data are presented as numbers with percentages in round parentheses and continuous data are presented as mean with standard deviation in round parentheses

**Suppl. table 1: Maternal characteristics comparison between the early and late complementary feeding group.**

| **Maternal characteristics** | ***Early (n=83)*** | ***Late (n=82)*** |
| --- | --- | --- |
| Gestational duration (days) | 190 (±16) | 191 (±14) |
| Age mother at birth (years) | 33 (±5) | 33 (±7) |
| BMI before pregnancy | 24 (±5) | 24 (±5) |
| Multiple birth | 32 (39%) | 26 (32%) |
| Cesarean delivery | 72 (87%) | 78 (95%) |
| Praeclampsia | 9 (11%) | 8 (10%) |
| Gestational diabetes | 3 (4%) | 3 (4%) |
| Maternal Education | | |
| primary education | 30 (36%) | 23 (28%) |
| secondary education | 10 (12%) | 18 (22%) |
| tertiary education | 31 (37%) | 22 (27%) |
| Maternal smoking habit | | |
| before pregnancy | 19 (23%) | 14 (17%) |
| during pregnancy | 3 (4%) | 1 (1%) |
| after pregnancy | 1 (1%) | 2 (2%) |
| always | 9 (11%) | 13 (16%) |
